# Hemoglobin scavenger receptor CD163 as a potential biomarker of hemolysis-induced hepatobiliary injury in sickle cell disease

**DOI:** 10.1152/ajpcell.00386.2023

**Published:** 2024-04-29

**Authors:** Tomasz W. Kaminski, Ayyanar Sivanantham, Anna Mozhenkova, Ashley Smith, Ramakrishna Ungalara, Rikesh K. Dubey, Bibhav Shrestha, Corrine Hanway, Omika Katoch, Jesús Tejero, Prithu Sundd, Enrico M. Novelli, Gregory J. Kato, Tirthadipa Pradhan-Sundd

**Affiliations:** ^1^Thrombosis and Hemostasis Program, Versiti Blood Research Institute, Milwaukee, Wisconsin, United States; ^2^Transfusion Medicine, Vascular Biology and Cell Therapy Program, Versiti Blood Research Institute, Milwaukee, Wisconsin, United States; ^3^Department of Medicine, Medical College of Wisconsin, Milwaukee, Wisconsin, United States; ^4^Pittsburgh Heart, Lung and Blood Vascular Medicine Institute, University of Pittsburgh School of Medicine, Pittsburgh, Pennsylvania, United States; ^5^Division of Hematology/Oncology, Department of Medicine, University of Pittsburgh School of Medicine, Pittsburgh, Pennsylvania, United States

**Keywords:** CD163, hemoglobin clearance, hemolysis, HO-1, sickle cell disease

## Abstract

Sickle cell disease (SCD)-associated chronic hemolysis promotes oxidative stress, inflammation, and thrombosis leading to organ damage, including liver damage. Hemoglobin scavenger receptor CD163 plays a protective role in SCD by scavenging both hemoglobin-haptoglobin complexes and cell-free hemoglobin. A limited number of studies in the past have shown a positive correlation of CD163 expression with poor disease outcomes in patients with SCD. However, the role and regulation of CD163 in SCD-related hepatobiliary injury have not been fully elucidated yet. Here we show that chronic liver injury in SCD patients is associated with elevated levels of hepatic membrane-bound CD163. Hemolysis and increase in hepatic heme, hemoglobin, and iron levels elevate CD163 expression in the SCD mouse liver. Mechanistically we show that heme oxygenase-1 (HO-1) positively regulates membrane-bound CD163 expression independent of nuclear factor erythroid 2-related factor 2 (NRF2) signaling in SCD liver. We further demonstrate that the interaction between CD163 and HO-1 is not dependent on CD163-hemoglobin binding. These findings indicate that CD163 is a potential biomarker of SCD-associated hepatobiliary injury. Understanding the role of HO-1 in membrane-bound CD163 regulation may help identify novel therapeutic targets for hemolysis-induced chronic liver injury.

## Graphical abstract

**Figure:**
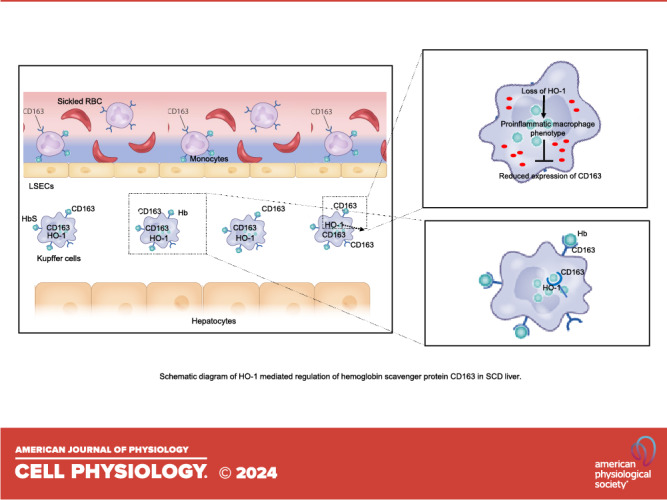

## INTRODUCTION

Sickle cell disease (SCD) is caused by a homozygous mutation in the β-globin gene, which leads to erythrocyte sickling, vasoocclusion, and intense hemolysis (1). SCD-induced hemolysis and accumulation of hemoglobin (2), heme, and iron (3) promotes both acute and chronic liver injury (1, 4–9) that affect up to 10–40% of SCD patients (10–15). However, therapeutic approaches to prevent liver injury in SCD are limited, and the mechanism promoting progressive liver injury in SCD remains poorly understood. Unfortunately, the reported incidence of liver complications has increased with the growing life expectancy of patients with SCD (16).

Previously, we have shown that SCD mice manifest hemolysis-induced iron-heme-hemoglobin accumulation (2, 3) and hepatobiliary injury under baseline conditions (17–19). Cell-free hemoglobin (Hb/HbS) released after intravascular red blood cell (RBC) hemolysis is scavenged by plasma haptoglobin, which chaperones it to the liver for hemoglobin scavenger receptor CD163 (20, 21)-dependent clearance by macrophages (hepatic Kupffer cells) (22, 23). However, chronic hemolysis in SCD results in depletion of plasma haptoglobin, leading to HbS clearance in the liver through a relatively less efficient process involving direct binding of Hb to CD163 on macrophages (22, 24–26). Thus, CD163 plays a pivotal role in scavenging hemoglobin-haptoglobin as well as cell-free Hb. A limited number of studies in the past have shown a positive correlation of CD163 expression with poor disease outcomes in patients with SCD (27–29). However, the role and regulation of CD163 in specifically regulating hepatobiliary injury in SCD have not yet been fully elucidated.

The transmembrane scavenger receptor CD163 is expressed exclusively on macrophages and monocytes as a membrane-bound form (30–34), which can be shed from the macrophage surface by inflammation-inducible TACE/ADAM17 enzyme, releasing the soluble CD163 in the blood (35). Whereas soluble CD163 has been previously associated with various disease pathophysiologies (28–31), the regulation of membrane-bound CD163 is not completely understood. This study aimed to investigate the regulation of membrane-bound CD163 in SCD liver using both a humanized mouse model and liver biopsy samples from patients. Here, we show that chronic liver injury in SCD patients is associated with elevated levels of membrane-bound hepatic CD163 in Kupffer cells and monocytes. SCD-induced chronic hemolysis (with the attendant increases in heme, hemoglobin, and haptoglobin) elevates CD163 expression in the liver of SCD mice. Mechanistically we show that heme oxygenase 1 (HO-1) positively regulates hepatic CD163 expression independent of nuclear factor erythroid 2-related factor 2 (NRF2) signaling. We further demonstrate that the interaction between CD163 and HO-1 is not dependent on hemoglobin binding of CD163. This study identifies CD163 as a potential biomarker of hemolysis-induced hepatobiliary injury in SCD. Thorough understanding of the HO-1-mediated signaling pathways that control membrane-bound CD163 expression will be useful in developing novel therapeutic approaches to treat hemolysis-induced tissue injury in SCD and other hemolytic diseases.

## METHODS

### Animals and Treatment

Townes SCD mice [SS, homozygous for Hba^tm1(HBA)Tow^, homozygous for Hbb^tm2(HBG1,HBB*)Tow^] and nonsickle control mice [AS, homozygous for Hba^tm1(HBA)Tow^, compound heterozygous for Hbb^tm2(HBG1,HBB*)Tow^/Hbb^tm3(HBG1,HBB)Tow^] (36) were obtained from the Jackson Laboratory (Bar Harbor, ME) and housed in a specific pathogen-free animal facility at the Medical College of Wisconsin and University of Pittsburgh animal facilities. The Institutional Animal Care and Use Committees at the Medical College of Wisconsin and University of Pittsburgh approved all animal experiments. Five or more mice were assessed at all given time points. Three or more male and female mice were used in all the experiments. Equal numbers of males and females were always used in these experiments, and unless otherwise stated all the mice used were 3 mo old. Although the majority of the mice used for this study were bred at the University of Pittsburgh, a portion of our mice were bred at the Medical College of Wisconsin to ensure the study’s completion. We recognize this circumstantial limitation linked to the study.

All animal experiments were approved by the Institutional Animal Care Committee at the University of Pittsburgh and the Medical College of Wisconsin under AUA-20087570 and AUA00008293, respectively. All the mice used were 25.0–28.0 g and were housed under controlled conditions of temperature (21–23°C), humidity (55%), and light (14:10-h light-dark regimen). The animals had free access to food (Lab diet 5LOD irradiated) and tap water ad libitum. Animals were housed in polypropylene cages (not more than 5 per cage) containing sterile paddy husk (PJ Murphy) as bedding and domes (Bio-Serv) and were regularly checked for pain or any discomfort. Mice were regularly checked for the symptoms of pain and distress like lethargic, shaking, hunched posture, droopy eyelids, red tears, loss of appetite, abdominal swelling, and retreating to a corner. Mice exhibiting these symptoms were excluded from the study. After reaching the end point of the experiment, ketamine hydrochloride (100 mg/kg) + xylazine (50 mg/kg) was administered intraperitoneally as anesthesia, and liver and blood were collected; after that, mice were euthanized by cervical dislocation under anesthesia.

#### Iron dextran treatment.

Control and SCD mice were administered 10 doses (1 dose every second day) of 100 mg/kg body wt iron dextran (Sigma D8517-25ML) intraperitoneally. Mice were euthanized after 3 wk, and liver and blood samples were collected for further analysis.

#### CD163 blocking.

CD163 activity was blocked with Antigenic Blocking Peptide CD163 N-epitope (FabGennix), which was administered subcutaneously at the dose of 1 mg/kg mouse body weight or as mentioned in the text. The peptide was dissolved in a warmed physiological saline solution and injected with insulin syringes with the ultrafine needle (BD, United States) into an immobilized mouse. The mouse was monitored for 60 min after the injection. The mice were euthanized 72 h post injection, and blood and liver were collected.

For competitive binding assay, the mice were injected with Antigenic Blocking Peptide CD163 N-epitope (FabGennix) via tail vein (iv) with three different doses of the blocker: 0.1 mg/kg, 1 mg/kg, and 3 mg/kg. After 60 min, all mice were administered 10 µM oxyhemoglobin (oxyHb) by tail vein route of injection. After 3 h mice were euthanized, and blood was collected from vena cava on sodium citrate as an anticoagulant. Blood was split into two tubes, and one tube was centrifuged (3,500 *g*, 15 min) to obtain platelet-poor plasma. Then, heme assay was performed as per the manufacturer’s instructions (heme assay kit; Abcam ab272534) on the whole blood and plasma.

#### Oxyhemoglobin treatment.

OxyHb (10 μm/kg) was injected intravenously by the tail vein route. Mice were euthanized after 6 h, and liver and blood samples were collected for further analysis.

#### LPS treatment.

Mice were administered 0.1 μg/kg LPS intravenously as described previously (37). Mice were euthanized after 3 h, and liver and blood samples were collected for further analysis.

#### Hypoxia-reperfusion treatment.

Control (AS) and SCD (SS) mice housed in their standard cages were transferred into a hypoxia chamber (BioSpherix) fitted with a ProOx 110 gas controller (BioSpherix) in line with a high-pressure double-gauge mixed gas primary nitrogen regulator fitted onto a vaporized nitrogen source. Mice were exposed to 7% oxygen for 2 h (hypoxia) and returned to room air (reoxygenation) for 1 h. Mice were euthanized, and liver and blood samples were collected for further analysis.

#### Clodronate-liposome treatment.

A high-dose clodronate-liposome treatment (100–150 μL/mouse) was used to deplete hepatic Kupffer cells. Mice were euthanized 24–72 h post injection.

### Proximity Ligation Assay

The method of tissue fixation and permeabilization is described in *Tissue Immunofluorescence*. Proximity ligation assay (PLA) assays were performed according to the manufacturer’s instructions (Sigma). For the visualization, Duo link in situ detection reagent red was used (Sigma) and nuclei were visualized with DAPI. Images of confocal slices were acquired with a Nikon A1 spectral confocal microscope. Control experiments were performed with just one primary antibody in the incubation. The signal was quantified with ImageJ.

### Western Blot

Immunoblotting was performed as described elsewhere (38). The primary antibodies used in this study are available in Supplemental Table S1. Membranes were washed five times for 5 min each in Tris-buffered saline-Tween 20 (TBST) before being probed with horseradish peroxidase (HRP)-conjugated secondary antibodies (1:5,000 diluted in 5% milk; Santa Cruz Biotechnology) for 1.5 h at room temperature. Membranes were washed three times for 10 min each in TBST and visualized with the Enhanced Chemiluminescence System (GE Healthcare). Supplemental Table S1 lists all the antibodies used for Western blotting.

### Heme Assay

Heme assay was performed as per the manufacturer’s instructions (heme assay kit; Abcam ab272534). Briefly, liver tissue was homogenized as directed. The homogenates were centrifuged, and heme levels were measured for each sample as per the manufacturer’s instructions. A similar method was used for plasma heme assay.

### Scanning Electron Microscopy

Whole liver was collected from control (AS), SCD, and SCD + clodronate mice for scanning electron microscopy (SE) imaging. Slices of whole liver were fixed in 2.5% glutaraldehyde in PBS (pH 7.4) for 10 min. Tissue samples were washed thoroughly in PBS for 15 min. Tissues were then fixed in 1% osmium tetroxide (OsO_4_) in PBS for 60 min. Samples were dehydrated with different concentrations of ethanol (30%, 50%, 70%, 90%) for 15 min and then critical point dried. Samples were visualized with a Field Emission Scanning Electron Microscope (JEOL JSM6335F) at a magnification of ×10,000–30,000.

### Serum Biochemistry

Aspartate aminotransferase (AST) and alanine aminotransferase (ALT) were measured in serum samples taken before euthanasia. Serum biochemistry was measured by automated testing in the Clinical Chemistry Division, University of Pittsburgh School of Medicine.

### Tissue Immunofluorescence

Tissue samples were frozen in OCT compound (Sakura 4583) on dry ice and stored at −80°C. Cryopreserved samples were cut into 5-µm sections, washed in PBS, and then fixed in 2% paraformaldehyde for 30 min. After washing, slides were washed with PBS and permeabilized with 0.1% Triton X-100 in PBS for 20 min at room temperature. Samples were washed three times with PBS and then blocked with 2% goat serum in 0.1% Tween 20 in PBS (PBST) for 30 min at room temperature. Antibodies were diluted in 2% goat serum-PBST and incubated at 4°C overnight. Primary and secondary antibodies used are available in the Supplemental Material. Images were taken on a Nikon A1 spectral confocal microscope. Supplemental Table S2 lists the antibodies used for immunohistochemical (IHC) assays.

### Coimmunoprecipitation Assay

Coimmunoprecipitation of TLR9 with HO-1 and CD163 was performed with the Pierce Direct IP kit (ThermoFisher Scientific, Illinois) per the manufacturer’s instructions. Briefly, anti-TLR9 and normal mouse IgG were each cross-linked with AminoLink Plus Coupling Resin in a rotator that goes from end to end for 120 min at room temperature. The compounds were centrifuged in a spin column to fix the antibody-resin mix. After washing with quenching buffer, sodium cyanoborohydride solution, 1× coupling buffer, and wash solution, the immobilized antibodies were ready for coimmunoprecipitation. Liver tissue was lysed with immunoprecipitation (IP) lysis buffer. Protein lysates were precleared with Control Agarose Resin to remove nonspecific combinations. For immunoprecipitation, the antibody-cross-linked resin was washed with IP wash buffer three times and added to protein lysate. The immunoprecipitation reaction was performed in a rotator at 4°C overnight. After that, immunoprecipitation products were eluted with elution buffer and analyzed with immunoblotting technique.

### mRNA Isolation and Real-Time Polymerase Chain Reaction

mRNA was isolated and purified from livers of AS and SCD mice (*n* = 3/group). mRNA was isolated with TRIzol (Invitrogen). RT-PCR was performed as described elsewhere (17, 39). Changes in target mRNA were normalized to GAPDH and 18S mRNA for each sample and presented as fold change over the average of the respective control group. Each sample was run in triplicate. Supplemental Table S3 lists all the primer sequences used for qRT-PCR analysis.

### siRNA Treatment in Hep3B Cells

The Hep3B human hepatoma cell line, which was obtained from ATCC (Manassas, VA), was transfected according to the manufacturers’ protocol. Briefly, Hep3Bs grown in Eagle’s minimal essential medium (ATCC) with 10% fetal bovine serum (Atlanta Biologicals, Lawrenceville, GA) were seeded onto six-well plates and transiently transfected with validated human HO-1 siRNA or negative control siRNA 1 (Ambion, Inc., Austin, TX) at a final concentration of 25 nmol/L in the presence of Lipofectamine MAX reagent (Invitrogen, Carlsbad, CA), as per the manufacturer’s instructions. The cells were harvested 72 h after transfection for RNA or protein extraction.

### Chromatin Immunoprecipitation Assay

The chromatin immunoprecipitation (ChIP) assay was conducted with the SimpleChIP Plus Sonication Chromatin IP Kit (no. 56383, Cell Signaling Technology), following manufacturer’s instructions. Briefly, 25 mg of tissue was chopped with a sterile razor blade and subsequently incubated with a 1% formaldehyde + protease inhibitor cocktail (PIC) mixture for 10 min at room temperature. After removal of the formaldehyde, tissue was suspended in 1 mL of 1× ChIP Sonication Cell Lysis Buffer + PIC for chromatin preparation. Nuclei were prepared, and the chromatin was sonicated with a Bioruptor Pico sonication device (Diagnode, Denville, NJ) with a 30 s on/30 s off cycle for 8 min. The collected supernatant was immunoprecipitated by incubates at 4°C for 12–16 h with 2 µg of anti-NRF2 antibody. The immunocomplexes were spun for 2 h at 4°C with 30 µL ChIP-Grade Protein G Magnetic Beads, followed by three 5-min washes with low-salt wash buffer and one with high-salt wash buffer. ChIP elution buffer was used to elute the chromatin for 30 min at 65°C with moderate vortex mixing (1,200 rpm). Cross-links were broken with 5 M NaCl and proteinase K for 2 h at 65°C. The samples were then treated with RNase A for 1 h at 37°C. Finally, ChIP DNA was purified and measured by qPCR.

### Quantification of CD163 Level by ELISA

The Mouse CD163 SimpleStep ELISA Kit (Abcam ab272204) was employed to assess the level of CD163, following the manufacturer’s protocol. Initially, 50 µL of all samples (liver lysate) or standards was dispensed into appropriate wells, followed by the addition of 50 µL of CD163 antibody cocktail to each well. The plate was then sealed and incubated for 1 h at room temperature with shaking. After the incubation period, the wells underwent three washes with a wash solution. Subsequently, the TMB substrate solution was added to the plate, and after a 10-min incubation with the substrate the enzymatic reaction was terminated. Finally, the absorbance of the resultant color was measured at 450 nm with a Tecan Infinite M200Pro plate reader.

### Binding Affinity of CD163 with Hba and HO-1 by Sandwich ELISA

In this study, we examined the binding affinity of CD163 with Hba and HO-1 post administration of CD163 blocker or oxyHb. Liver lysate obtained from SCD mice was treated with 3 mg/mL of CD163 blocker or 10 µM oxyHb. After a 30-min incubation period, the samples were applied to a plate coated with CD163-specific antibodies. Additionally, capture antibodies targeting Hba and HO-1, or control IgG, along with an HRP-conjugated secondary antibody, were introduced to the plate. After a 1-h incubation, the wells were washed three times with a wash solution. Subsequently, the TMB substrate solution was added to the plate, and after a 10-min incubation with the substrate the enzymatic reaction was halted. Finally, the absorbance of the resulting color was measured at 450 nm with a Tecan Infinite M200Pro plate reader.

### Statistical Analysis

All comparisons between two groups were deemed statistically significant by unpaired two-tailed Student’s *t* test if *P* < 0.05 or *P* < 0.01. When more than two groups were compared, statistical analysis was done by one-way and two-way ANOVA with Bonferroni correction. Calculations were performed with Prism version 7.0a (GraphPad Software). All in vitro studies are either a compilation of three independent experiments or representative of at least three independent experiments. Error bars represent standard deviation.

### Human Subjects

This study was approved by the local Institutional Review Board (IRB-U.Pitt) and conducted in accordance with the Declaration of Helsinki and NIH guidelines for using human specimens. Deidentified needle biopsy specimens of liver from SCD patients and age-matched healthy control human subjects were retrospectively reviewed for associated pathology and liver disease. Samples were obtained for light microscopy by standard procedures (17).

## RESULTS

### Hemolysis-Induced Accumulation of Hemoglobin-Heme-Iron Promotes the Expression of Membrane-Bound CD163 in SCD

**Figure 1.**
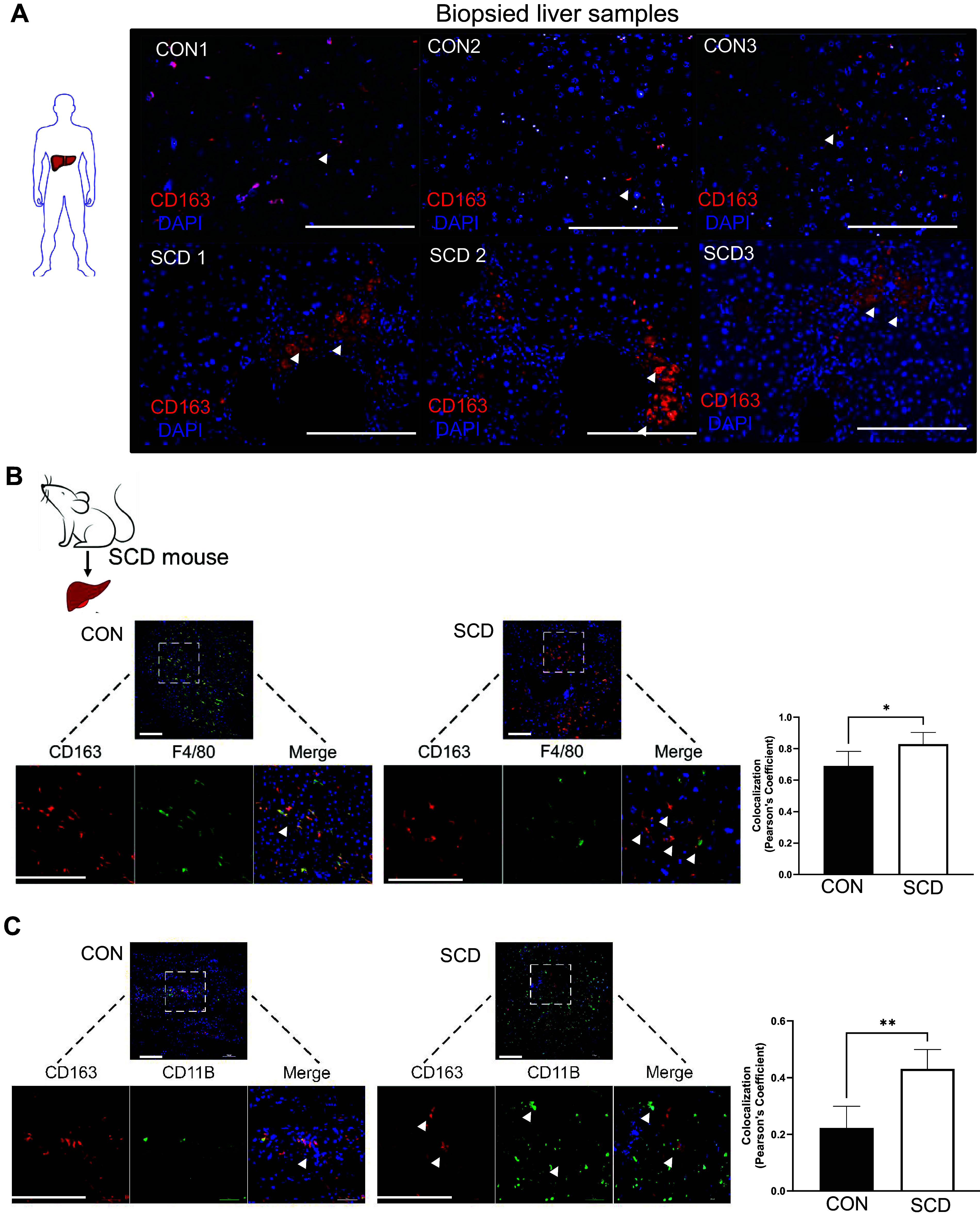
Hemoglobin scavenger receptor CD163 is associated with hepatobiliary injury in sickle cell disease (SCD). *A*: representative images of immunofluorescence (IF) analysis of SCD patient biopsied liver samples show abundant CD163-positive cells. *B*, *left*: representative IF images (merged and single channels) of colocalization assay show higher percentage of CD163 colocalized with positive F4/80 staining of Kupffer cells in SCD mouse liver. *Right*: bar graph depicts the Pearson’s coefficient of colocalization in control (CON) and SCD mouse liver. *C*, *left*: representative IF images (merged and single channels) of colocalization assay show higher percentage of CD163 colocalized with positive CD11b staining of monocytes in SCD mouse liver. *Right*: bar graph depicts the Pearson’s coefficient of colocalization in control and SCD mouse liver. The error bars represent SD. Arrowheads indicate liver sinusoidal endothelial cells. *P < 0.05, **P < 0.01. Scale bars, 50 µm.

To assess the role of CD163 in hemolysis-induced hepatobiliary injury in SCD, we first analyzed the hepatic expression of CD163 (membrane-bound CD163) in SCD patient biopsied liver samples. Immunofluorescence (IF) of CD163 showed significant upregulation of CD163 in hepatic Kupffer cell-like structures in SCD patients compared to healthy control human liver samples (Fig. 1*A*).

**Figure 2.**
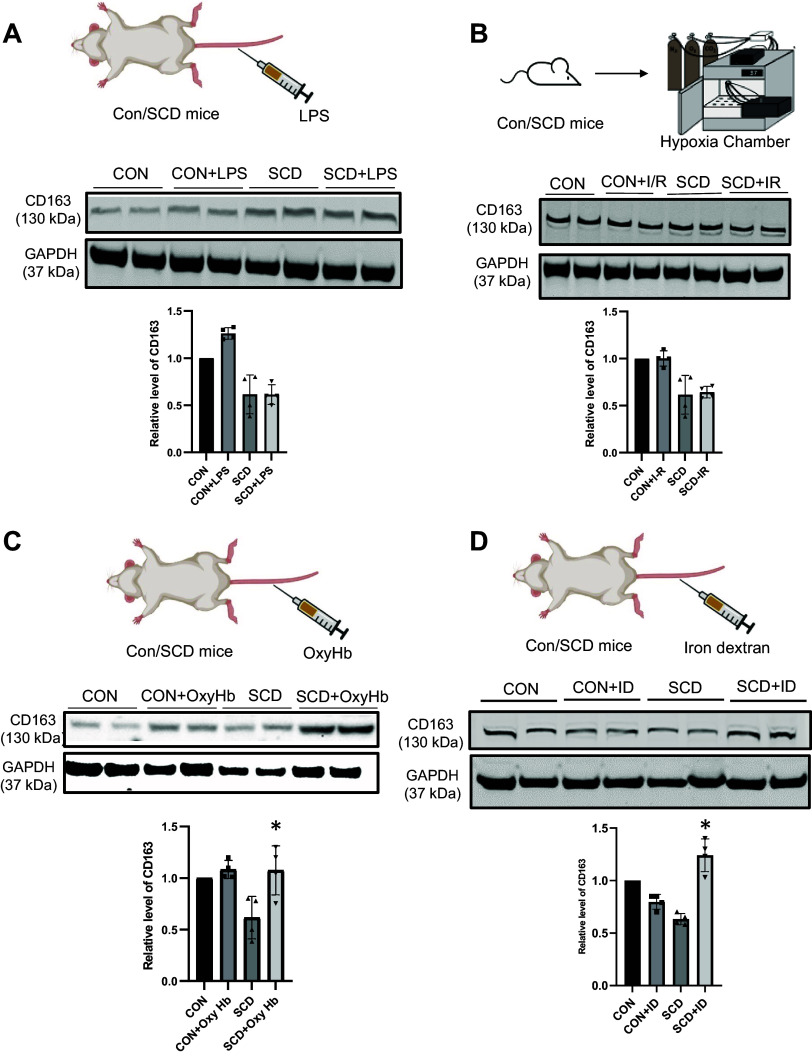
CD163 expression is regulated by hemolysis associated with sickle cell disease (SCD). *A* and *B*, *top*: the schemes of LPS (*A*) and hypoxia (*B*) [in SCD and littermate control (CON) mice]. *Bottom*: representative Western blot micrographs show unchanged levels of CD163 in the liver of SCD and control mice after LPS or hypoxia induction. Densitometric analysis of CD163 protein in each condition: the error bars represent SD. *C, top*: scheme of treatment SCD and control mice with intravenous injection of 10 µM oxyhemoglobin (oxyHb) for hemolysis induction. *Bottom*: representative Western blot micrograph and densitometric analysis show increased levels of CD163 in the liver of SCD mice after oxyHb treatment. *D, top*: scheme of SCD and control mouse treatment with 100 mg/kg body wt of iron dextran (ID). Bottom: representative Western blot micrograph and densitometric analysis reveal higher levels of CD163 in the liver of SCD mice after ID treatment compared to other treatments. The error bars represent SD. *P < 0.05. Figure created with BioRender.com.

To further analyze the effect of SCD-induced hemolysis in SCD, we used the Townes SCD mouse model. IF revealed that CD163 is predominantly expressed in hepatic monocytes and Kupffer cells in both control and SCD mice (Fig. 1, *B* and *C*). Quantification of colocalization revealed significant upregulation of CD163 in the hepatic monocytes and Kupffer cells in SCD mouse liver (Fig. 1, *B* and *C*). Next, we investigated the factors that might influence CD163 expression in the SCD mouse liver. Interestingly, whereas administration of LPS (0.1 μg/kg; Fig. 2*A*; bar graph shows densitometric analysis) or hypoxia (Fig. 2*B*; bar graph shows densitometric analysis) did not influence CD163 expression, increase in hepatic oxyhemoglobin (Fig. 2*C*; bar graph shows densitometric analysis), and iron content (Fig. 2*D*; bar graph shows densitometric analysis) caused significant enrichment of CD163 in the liver of SCD mice compared to littermate control mice. Taken together, our data suggest that CD163 increases in SCD mouse and patient liver because of hemolysis-induced hepatic accumulation of hemoglobin-heme and iron.

### Loss of CD163 Promotes Hemolysis-Induced Hepatobiliary Injury in SCD Mouse Liver

As CD163 expression positively correlated with hemolysis, which is a main pathological factor in SCD, we hypothesized that the increased expression of CD163 in SCD liver would protect against hemolysis-induced hepatobiliary injury. To establish the protective role of CD163 in SCD-induced hepatobiliary injury, we depleted CD163 in the SCD liver by two different approaches.

**Figure 3.**
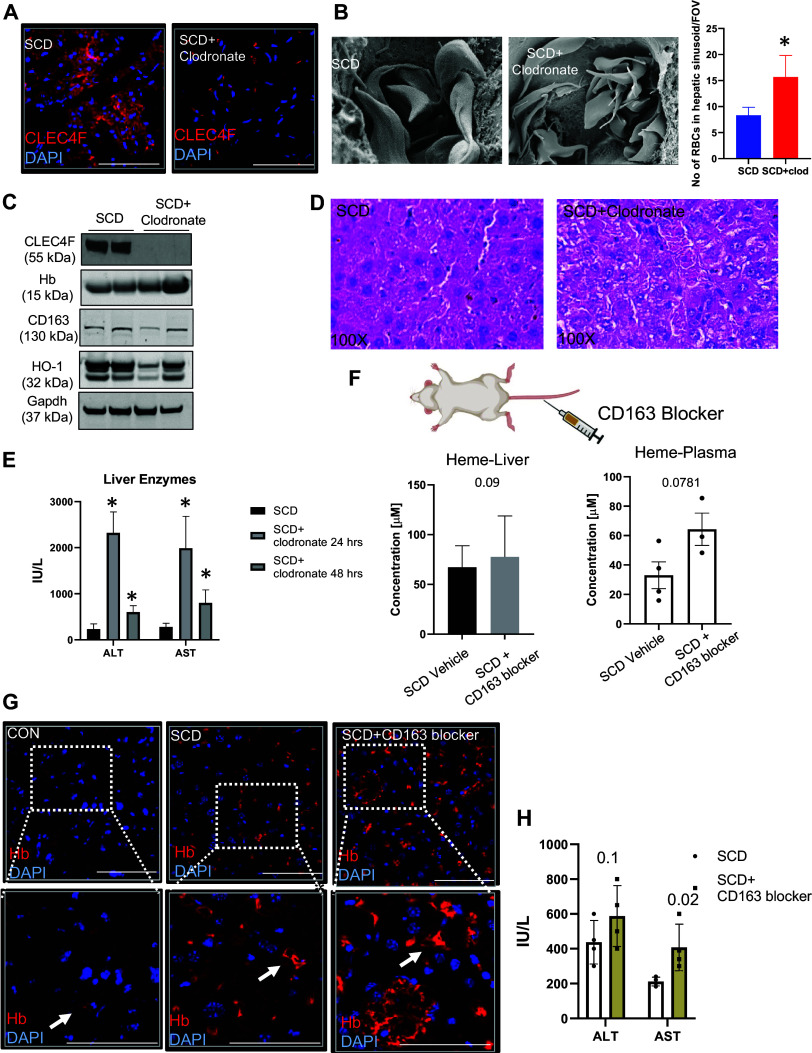
Loss of CD163 promotes hemolysis-induced hepatobiliary injury in the sickle cell disease (SCD) mouse liver. *A*: representative immunofluorescence staining shows strong reduction of Kupffer cell marker CLEC4F in the SCD mice liver post clodronate-liposome treatment (*right*) compared to nontreated SCD mice (*left*). Scale bars, 50 µm. *B*, *left*: representative scanning electron microscopy (SE) images show increased presence of hepatic hemoglobin and total number of erythrocytes sequestered in hepatic sinusoids in SCD mice liver after Kupffer cell depletion. *Right*: statistical analysis reveals increased numbers of red blood cells (RBCs) sequestered in the hepatic sinusoids per field of view (FOV). *C*: Western blot analysis of CD163 shows reduced expression post macrophage depletion in SCD mouse liver. Kupffer cell marker CLEC4F expression is reduced post clodronate treatment. Kupffer cell depletion also caused hepatic hemoglobin (Hb) accumulation and reduced expression of heme oxygenase-1 (HO-1) in SCD mice post clodronate treatment compared to untreated SCD mice. *D*: representative hematoxylin and eosin (H&E) staining shows increased hepatic vasoocclusion and several entrapped RBCs in the liver of SCD mice post clodronate treatment. Magnification: ×100. *E*: biochemical analysis of the liver injury markers shows that clodronate-liposome treatment leads to significant (∼10-fold) increase in concentrations of aspartate aminotransferase (AST) and alanine aminotransferase (ALT) 24 h post treatment compared to vehicle-treated mice. This increase attenuated after 48 h post treatment, but the levels of markers remained elevated compared to control mice. *F*: ELISA shows that blockage of CD163 activity by an antigenic blocking peptide resulted in increased levels of heme in plasma with also a slight elevation in the liver of SCD mice. *G*: representative IF images showing hemoglobin accumulation in the liver of control mouse, SCD mouse and SCD mouse treated with CD163 blocker. Arrow indicates hemoglobin positive cells. Scale bars, 50 µm. *H*: liver injury biomarkers ALT and AST were elevated in SCD mouse serum after CD163 blocking compared to untreated mice. The error bars represent SD. Scale bars, 50 μm. *P < 0.05. Figure created with BioRender.com.

As the liver resident macrophages (Kupffer cells) are the primary cells known to express CD163 (21), we first depleted hepatic Kupffer cells with clodronate-liposome as previously described (40). We observed a strong reduction of Kupffer cell marker CLEC4F in the liver post clodronate-liposome treatment as expected (Fig. 3*A*). Kupffer cell depletion also led to an increase in total number of erythrocytes sequestered in hepatic sinusoids (as shown by SE in Fig. 3*B*; bar graph shows quantification). Interestingly, we found that the depletion of hepatic Kupffer cells in SCD mice by clodronate liposomes led to strong loss of CD163 (Fig. 3*C*), Hb, as well as heme oxygenase 1 (HO-1). Hematoxylin and eosin (H&E) staining showed increased entrapment of RBCs in SCD mice post clodronate treatment (Fig. 3*D*). We also confirmed exacerbated liver injury in this model as evidenced by the enhanced expression of serum markers of liver injury (ALT and AST) (Fig. 3*E*).

Additionally, we used a CD163 peptide blocker (previously uncharacterized) to examine the specific effects of blocking CD163 activity in the liver of SCD mice. Blocking CD163 activity led to a significant increase in serum and hepatic heme levels, confirming the loss of function of CD163 (Fig. 3*F*). As in the case of Kupffer cell depletion in the SCD mouse liver, we found enhanced accumulation of Hb in the SCD liver post CD163 depletion by IF (Fig. 3*G*). Serum markers of liver injury were also significantly upregulated (Fig. 3*H*) in SCD mice after blocking CD163, suggesting its role in protection against hemolysis-induced hepatobiliary injury in SCD.

**Figure 4.**
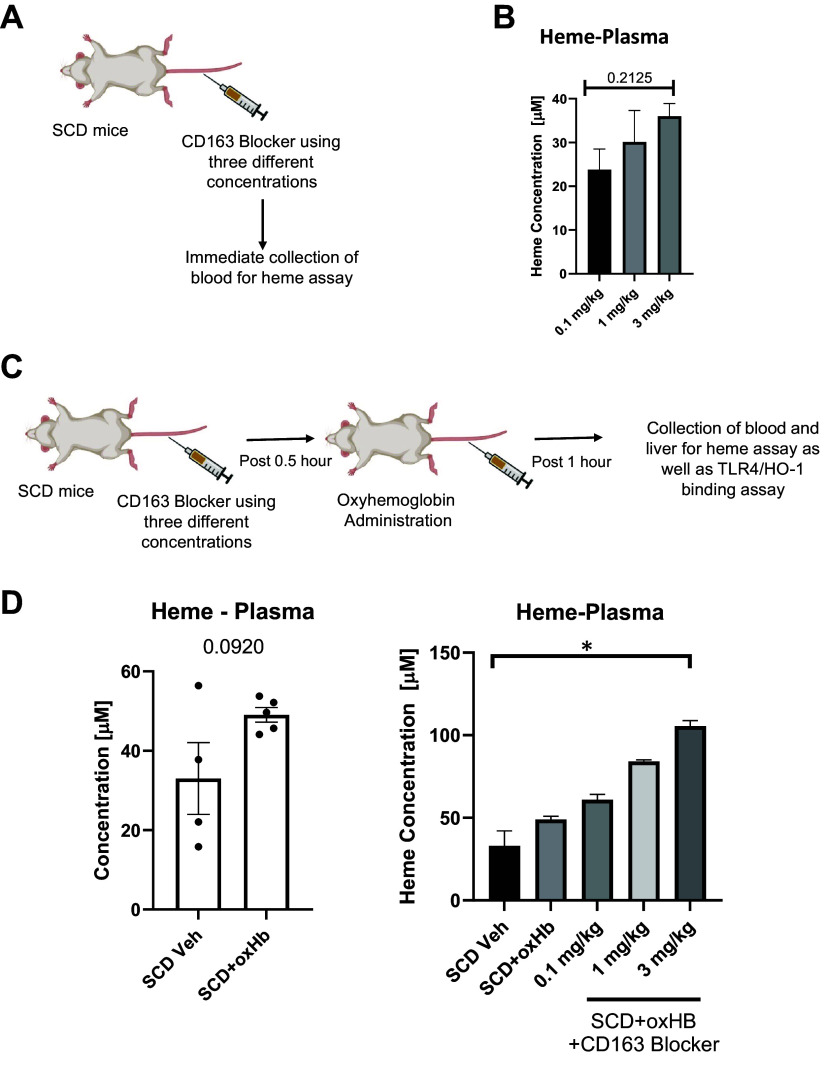
CD163 blocker competes with hemoglobin (Hb) for binding. *A*: schematic showing the experimental plan to analyze the effect of CD163 blocker in sickle cell disease (SCD) mouse liver. *B*: bar graph depicting the results of heme ELISA assay post administration of CD163 blocker in the plasma of SCD mice. *C*: schematic showing the experimental plan to examine the mode of action of the CD163 blocker used in blocking CD163-Hb binding. HO-1, heme oxygenase-1. *D*: bar graphs depicting the results of heme ELISA assay post CD163 blocker administration in the plasma of SCD mice. oxHb, oxyhemoglobin; Veh, vehicle. The error bars represent SD. *P < 0.05. Figure created with BioRender.com.

To further confirm the specificity of the CD163 blocker as well as to understand its mode of action, we performed the following set of experiments. We delivered three different concentrations (from lowest to highest) of CD163 blocker to SCD mice and then measured the amount of heme in their blood. Our hypothesis was that if CD163 blocker inhibited CD163 function, it would be unable to bind with Hb, resulting in an increase in total heme levels in the blood (Fig. 4, *A* and *B*). We also hypothesized that the increase in heme level would be positively correlated with the increasing concentration of the CD163 blocker utilized. As shown in Fig. 4*B*, we found that the amount of heme in the blood increased significantly after CD163 blocking, and it correlated positively with the increasing concentration of the blocker.

Next, to decipher the competition between CD163 and its natural substrate (Hb) and the therapeutic blocker, we used the following approach. We administered three different concentrations (from lowest to highest) of CD163 blocker to SCD mice, followed by a second administration of oxyhemoglobin 0.5 h later in the same mice, and then measured heme levels in the blood and liver within 1 h (Fig. 4*C*). Our hypothesis was that if the blocker and the natural substrate (Hb) compete for the same binding site, then adding the blocker first will not let Hb bind with CD163 anymore, resulting in significant increase in plasma heme level. As indicated in Fig. 4*D*, heme levels were found to be dramatically raised in both the blood and plasma of SCD mice, and it positively correlated with the concentration of the CD163 blocker utilized. Notably, the heme level post administration of blocker and oxyhemoglobin treatment was significantly higher than oxyhemoglobin treatment alone (Fig. 4*D*). Thus, we speculate that the CD163 blocker inhibits Hb-CD163 binding by competing for the same binding site. Collectively, our data also suggest that loss of CD163 activity further exacerbates hemoglobin-heme-iron-induced hepatobiliary injury in SCD.

### HO-1 Positively Regulates Hepatic CD163 Expression Independent of NRF2 Signaling

**Figure 5.**
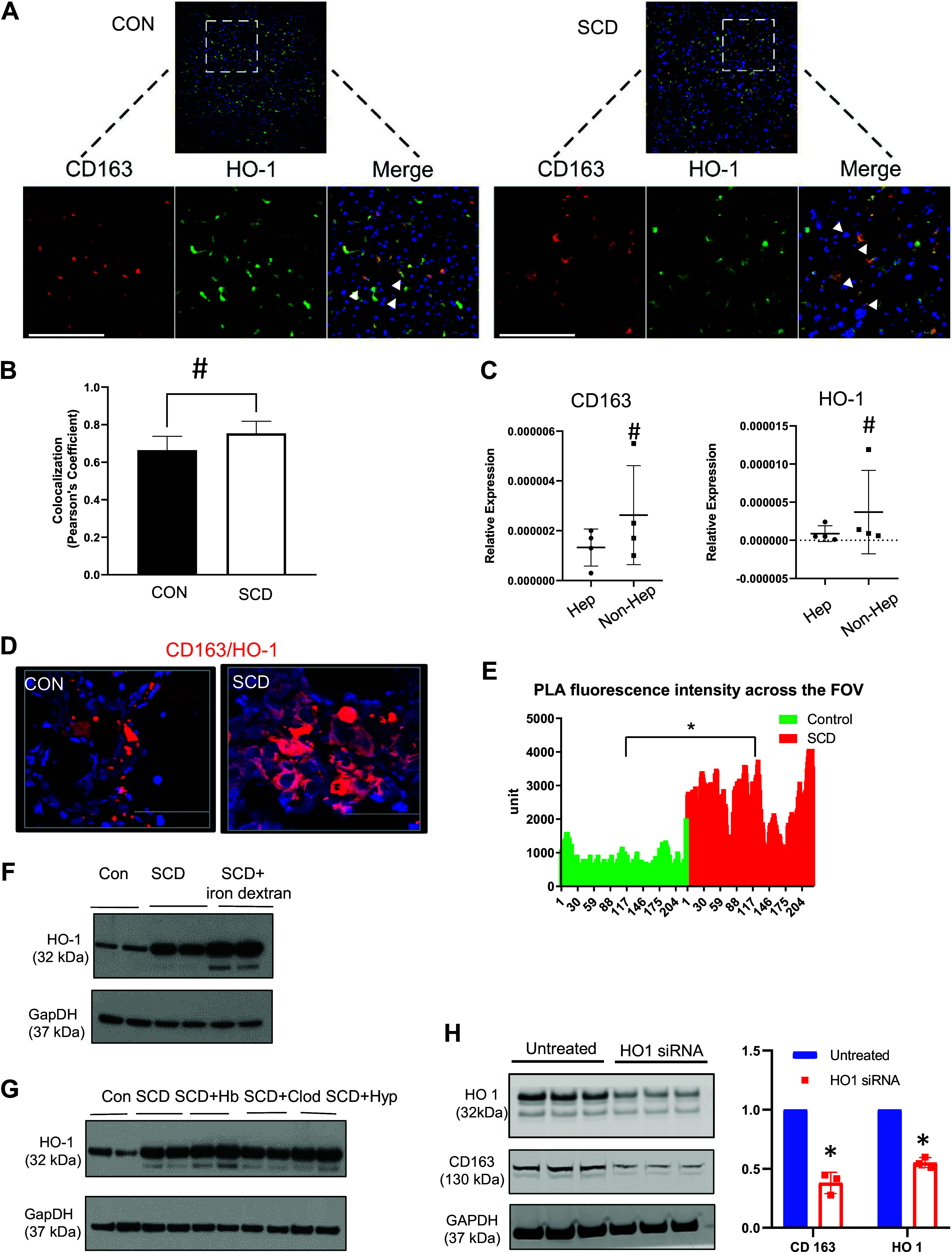
Heme oxygenase-1 (HO-1) positively regulates CD163 level in sickle cell disease (SCD) mouse liver. *A* and *B*: representative immunohistochemistry image (*A*) and colocalization analysis (*B*) show increased colocalization of CD163 and HO-1 in hepatic Kupffer cells of SCD mouse liver. CON, control. *C*: qRT-PCR analysis of hepatocytes and nonhepatocytes isolated from control mouse liver exhibits increased expression of both CD163 and HO-1 in the nonhepatocyte cell population. *D*: the representative images acquired during proximity ligation assay (PLA) show increased interactions between CD163 and HO-1 in SCD mice compared to littermate control mice. Scale bars, 50 µm. *E*: fluorescence intensity quantification of PLA demonstrating significantly higher intensity in the SCD group compared to control mice. FOV, field of view. *F* and *G*: series of Western blot micrographs (for the treatment pattern see Fig. 2) showing the levels of HO-1 in liver lysates of control mice, untreated SCD mice, and SCD mice after treatment with iron dextran (*F*) and treatments with clodronate and hemoglobin as well as undergoing hypoxic conditions (*G*). *H*, *left*: representative Western blot micrographs showing decreased levels of CD163 protein expression after HO-1 knockdown in Hep3B human hepatoma cell line compared with untreated cells. *Right*: densitometric analysis of micrographs shows significantly reduced levels of CD163 protein expression after HO-1 knockdown and confirms the efficiency of performed knockdown in control and SCD mouse liver tissue. *P < 0.05; #*P* < 0.06–0.08.

To understand the significance of membrane-bound CD163 in protecting against hemolysis-induced liver injury in SCD and to define the signaling pathway linked to membrane-bound CD163-mediated injury resolution, we analyzed the molecular mechanism behind the elevated CD163 expression in the SCD liver. Once internalized through the CD163 receptor, the hemoglobin/hemoglobin-haptoglobin complex is degraded by heme oxygenase 1 (HO-1) to release heme, carbon monoxide, and biliverdin/bilirubin (22, 41). As CD163 and HO-1 are intricately linked in hemoglobin trafficking and degradation, and Kupffer cell depletion led to reduced expression of both CD163 and HO-1, we examined whether HO-1 can regulate CD163 levels. We found colocalization of CD163 (Fig. 2*B*) and HO-1 (Fig. 5*A*, quantified in Fig. 5*B*) by IHC in both control and SCD mouse liver that was significantly increased in SCD mice. As hepatic Kupffer cells are the primary nonhepatocyte cell type in the liver parenchyma, we analyzed the mRNA expression of HO-1 and CD163 in hepatocytes and nonhepatocytes isolated from the liver of SCD mice. As shown in Fig. 5*C*, nonhepatocytes had significantly higher expression of both HO-1 and CD163.

To identify potential interactions between CD163 and HO-1, we first performed a proximity ligation assay in control and SCD mouse liver. Both control and SCD mouse liver showed a positive signal, which was increased significantly in SCD mice (Fig. 5*D*, quantified in Fig. 5*E*). Moreover, Western blot analysis showed that HO-1 displays a similar expression pattern post hemoglobin-heme-iron accumulation (Fig. 5, *F* and *G*). Next, we explored whether HO-1 can regulate CD163 expression in the liver. Remarkably, knocking down HO-1 in the Hep3B liver cell line caused significant reduction of CD163 protein expression (Fig. 5*H*, quantified).

**Figure 6.**
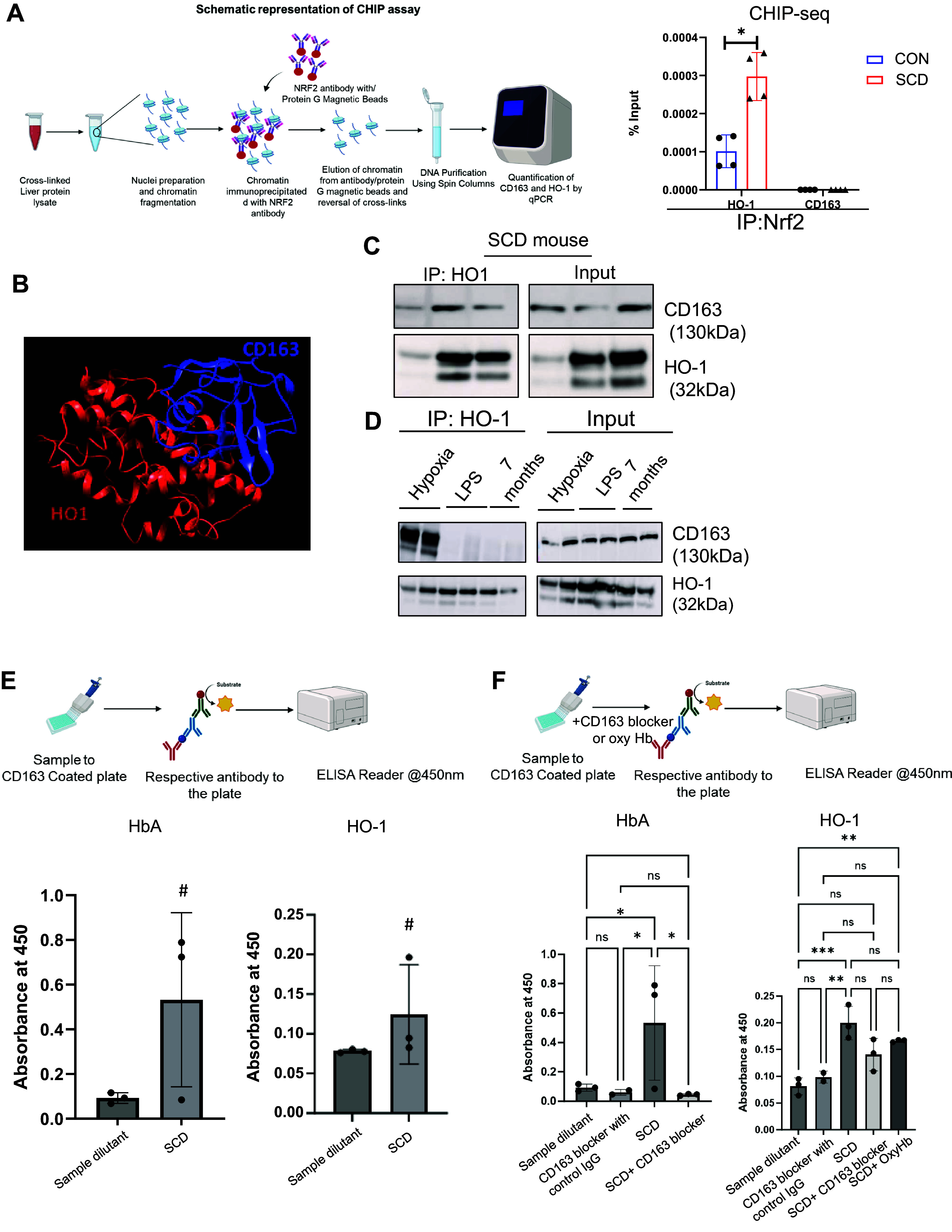
Heme oxygenase-1 (HO-1) positively regulates CD163 level independent of nuclear factor erythroid 2-related factor 2 (NRF2) signaling. *A, left*: schematic showing the experimental flow of chromatin immunoprecipitation (ChIP) assay to determine the binding between CD163-NRF2 as well as NRF2 and HO-1. *Right*: bar graph depicts that compared to control mice sickle cell disease (SCD) mice exhibit higher levels of NRF2 binding to the HO-1 gene in the liver. The error bars represent SD. IP, immunoprecipitation. *B*: bioinformatic predictive tool analysis suggesting the presence of a molecular basis for the binding of CD163 and HO-1 with TLR9. *C*: representative Western blot micrograph showing coimmunoprecipitation confirming complex formation between CD163 and HO-1 in SCD mouse liver. *D*: representative Western blot micrograph showing the effect of hypoxia, LPS, and aging in CD163 and HO-1 interaction in SCD mouse liver. *E*, *top*: schematic diagram depicting the steps of sandwich ELISA assay. *Bottom*: comparative analysis of absorbance suggests stronger binding affinity of CD163-hemoglobin (Hb) compared to CD163-HO-1. *F*: comparative analysis of absorbance values suggests that whereas CD163 blocker blocks CD163-Hb binding, no significant change was seen in CD163-HO-1 binding. Similarly, administration of oxyhemoglobin did not significantly change CD163-HO-1 binding. *P < 0.05; **P < 0.001; ***P < 0.005; #*P* < 0.06–0.08. ns, Not significant. Figure created with BioRender.com.

The transcription factor nuclear factor erythroid-derived-2-like 2 (NFE2L2), also known as NRF2, regulates a subset of downstream target genes including HO-1 (42–44). We hypothesized that HO-1 also regulates CD163 via NRF2 (45). To confirm the effect of NRF2 on CD163 expression, we performed a CHIP-seq analysis. Interestingly, CHIP-seq confirmed no binding/association between NRF2 and CD163 (Fig. 6*A*). NRF2-HO-1 binding was used as a positive control, and as shown in Fig. 6*A* the result confirmed that NRF2 can bind to HO-1. In addition, we found a higher relative enrichment of NRF-2 binding to HO-1 in SCD mice compared to littermate control mice. Altogether, these findings suggest that HO-1 regulates CD163 expression independent of NRF2 in the SCD mouse liver.

### Membrane-Bound CD163 Can Form a Complex with HO-1 Independent of Its Hemoglobin Binding Capacity

Since HO-1 regulates CD163 independently of NRF2, we hypothesized that HO-1 may regulate CD163 posttranslationally, independent of its Hb binding capacity. To confirm the possibility of an interaction between HO-1-and CD163, we used two different complementary approaches. First, to understand the interaction of HO-1 and CD163 we performed a bioinformatic analysis. The crystal structures of HO-1 (1N3U) and CD163 (6K0O) were retrieved from the Protein Data Bank (www.rcsb.org) and Uniprot. The potential interactions with HO-1 with CD163 were carried out with the pyDockWEB platform (https://life.bsc.es/servlet/pydock). Docked complex was visualized by the UCSF Chimera molecular visualization program. As shown in Fig. 6*B*, the algorithm predicted strong binding affinity of HO-1 with CD163. Second, to confirm the interaction between CD163 and HO-1 we performed a coimmunoprecipitation assay. HO-1 and CD163 formed a complex as shown in Fig. 6*C*.

Next, we examined the effect of hypoxia, inflammation, and aging, a common set of pathophysiology frequently linked to SCD, in regulating CD163-HO-1 interaction. We administered LPS in SCD mice to induce increased inflammation and injury. To induce oxidative stress in SCD mice we utilized hypoxia chambers (4 h of hypoxia followed by 2 h of normoxia). To compare the effect of aging we used 7-mo-old SCD mouse liver. We performed a coimmunoprecipitation assay between HO-1 and CD163 under each of these conditions. As shown in Fig. 6*D*, compared to control (SCD mouse liver at baseline) liver (Fig. 6*C*), oxidative stress caused an increase in CD163-HO-1 binding. However, aging as well as increased injury and inflammation led to a decrease in this binding.

Finally, as hemoglobin is the known natural substrate for CD163, we examined whether HO-1 competes with Hb for its binding or whether the CD163-HO-1 binding is independent of the Hb binding capacity of CD163. We performed a sandwich ELISA assay to further determine the binding dynamics of CD163 with Hb and HO-1, respectively. As shown in the schematic diagram in Fig. 6*E*, we added SCD mouse liver lysate in CD163-coated ELISA plates, followed by addition of Hb and HO-1 antibodies to the wells. Hb and CD163 binding appeared strongest with highest absorbance values. However, HO-1 showed comparatively weaker binding dynamics and absorbance values than those of Hb. Moreover, adding CD163 blocker to the wells before antibody administration blocked Hb binding to CD163 (Fig. 6*F*) but did not affect the CD163-HO-1 binding significantly. Similarly, adding oxyhemoglobin to the wells resulted in no significant changes in binding affinity between HO-1 and CD163 (Fig. 6*F*). Collectively, our data suggest that membrane-bound CD163 interacts with Hb as well as HO-1. Whereas binding with Hb is of strongest affinity, and promotes Hb clearance, HO-1 binding could lead to cytoprotective functions of the membrane-bound form of CD163 and is not dependent on Hb-CD163 complex formation.

## DISCUSSION

Over the last few decades, research on the multifaceted pathophysiology associated with HbS polymerization and hemolysis in SCD has led to the identification of many new therapeutic molecules (46–48). Previous studies in children and adults with SCD have shown increased soluble CD163 levels in the plasma that correlated with pulmonary hypertension and vasoocclusion. Along that line, we here show that membrane-bound CD163 can potentially be used as a novel therapeutic molecule/biomarker of SCD-related hepatobiliary injury. We confirm that elevated hemoglobin, heme, and iron levels stimulate CD163 expression in the SCD liver. Loss of CD163 expression or activity is associated with exacerbated liver injury in SCD patients due to accumulation of hemoglobin in the liver. Although previous studies have suggested LPS-induced inhibition of CD163 (31), we did not see any change in CD163 expression upon LPS treatment in the hepatic Kupffer cells. The differences can be attributed to differences in dosage, tissue-specific effects, or other unknown effects associated with increased hepatic hemoglobin level and need to be addressed in future.

**Figure 7.**
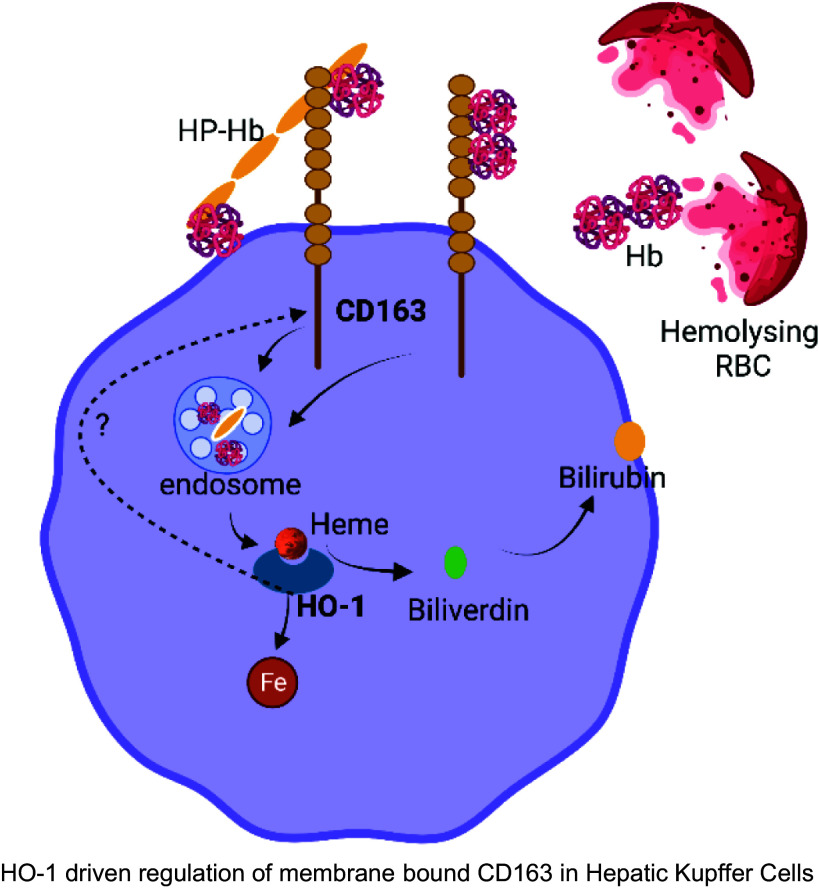
The CD163 and heme oxygenase-1 (HO-1) signaling pathway in sickle cell disease (SCD)-related liver complications. Schematic diagram depicting the HO-1-CD163 interaction in SCD mouse liver. Cell-free hemoglobin (Hb) released after intravascular hemolysis is scavenged by plasma haptoglobin (HP), which chaperones it to the liver for hemoglobin scavenger receptor CD163-dependent clearance by macrophages. Cell-free Hb on its own can also bind to CD163, albeit less efficiently. In both cases, it leads to endocytosis and degradation of Hb and the release of heme. HO-1 metabolizes heme to produce carbon monoxide, iron, and biliverdin. Along with its role in heme degradation, HO-1 also regulates CD163 expression in the hepatic Kupffer cells. Loss of HO-1 in the liver reduces CD163 expression, which can further impact the Hb-heme metabolism in SCD liver. RBC, red blood cell. Figure created with BioRender.com.

HO-1 is a crucial enzyme in the process of heme detoxification as it catalyzes the decomposition of heme into biliverdin, iron, and carbon monoxide (49, 50). Along with heme detoxification, HO-1 has cytoprotective, anti-inflammatory, and antioxidant properties (51, 52). Thus, the activation of HO-1 is a critical factor in safeguarding tissues and endothelium from oxidative stress and hemolysis. Previous studies have confirmed that HO-1 is upregulated in the SCD mouse liver to protect against hemolysis- and oxidative stress-induced tissue damage (3, 44, 53, 54). HO-1-mediated regulation of membrane-bound CD163 hints at an additional protective role of HO-1 in hemoglobin scavenging. Our findings are consistent with the previously recognized role of CD163 in HO-1/HMOX1 expression in macrophage cell lines (55, 56). Both CD163 and HO-1 have been linked to anti-inflammatory and wound healing properties (33, 57–60). Their expression is also dependent on endocytic trafficking (55) and IL10 (61, 62). Thus, although the effect of HO-1 on membrane-bound CD163 expression could be due to a direct interaction, other indirect effects through changes in iron/ferritin levels, HO-1-mediated endocytic trafficking, or IL10 regulation cannot be ruled out. We hypothesize that the regulation of CD163 by HO-1 might occur through the following two mechanisms (Fig. 7): *1*) Loss of HO-1 promotes accumulation of heme and thereby increases the proinflammatory status of the cells. Previous studies have shown that CD163 is negatively regulated by the proinflammatory status of the cells (63, 64). Thus, HO-1 can indirectly upregulate the three proteins via its proinflammatory action. *2*) Loss of HO-1 can change the polarity of hepatic Kupffer cells, which might affect the localization and stability of CD163.

A key finding of our study is NRF2-independent regulation of CD163 by HO-1. We found that NRF2 cannot transcriptionally regulate CD163 in a CHIP-seq assay. The Keap1-NRF2 signaling pathway controls the expression of many cytoprotective and antioxidant genes, including HO-1 (45). NRF2-independent roles of HO-1 are not very well understood. Our study highlights one such previously undescribed function of HO-1 in the SCD liver. As there are several other transcription factors needed for NRF2 dimerization and downstream signaling (65), future work will be needed to confirm whether there is any potential association of those transcriptional factors with HO-1 in the regulation of CD163. It will be very interesting to characterize the downstream effect of oxidative stress, inflammation, as well as aging on CD163-HO-1 binding.

Our study demonstrates that although membrane-bound CD163 has a higher binding affinity for Hb compared to HO-1, blocking or increasing Hb binding does not impact CD163-HO-1 interaction. This conclusion is supported by a comparative analysis of the binding affinities of CD163 with Hb and HO-1 with or without the blocker. Similarly, administration of oxyhemoglobin increased CD163-HO-1 interaction. Thus, we speculate that membrane-bound CD163 binds with HO-1 post Hb binding and internalization and binding with HO-1 might promote CD163 recycling. We also speculate that, whereas the main role of CD163 in SCD appears to be binding to and removal of cell-free Hb, the interaction with HO-1 also holds importance (particularly in SCD) and could enhance the cytoprotective effects of CD163 and its stability/endocytic recycling. Subsequent investigations should verify whether soluble CD163 exhibits comparable binding affinity as well as identifying the binding sites.

The study has a few limitations. First, inflammation in humans and mice activates different subsets of signaling pathways. Future work is needed to confirm the interaction of HO-1 and CD163 in hepatobiliary injury in patients with SCD. Second, although we see loss of expression of HO-1 and CD163 upon Kupffer cell depletion, the exact role of Kupffer cells in regulating the expression of these proteins needs to be determined. Third, the effect of HO-1 on soluble CD163 was not examined in this study. Finally, given the expanding interest in altering macrophage activity toward cancer, the potential of our findings might be beneficial to liver cancer treatment in patients with hemoglobinopathies (35). In summary, our finding of a protective role of CD163 in hemolysis-induced organ damage may inspire future investigations exploring the molecular mechanism underlying CD163-mediated hepatic Hb clearance not only in SCD but also in other hemolytic diseases.

## DATA AVAILABILITY

Data will be made available upon reasonable request.

## SUPPLEMENTAL MATERIAL

10.6084/m9.figshare.25533679 Supplemental Material: https://doi.org/10.6084/m9.figshare.25533679.

## GRANTS

This work was supported by National Institutes of Health (NIH), National Institute of Diabetes and Digestive and Kidney Diseases 1K01DK125617-01 (T.P.-S.), American Society of Hematology junior faculty scholar award (T.P.-S.), and Versiti start-up fund (T.P.-S.); National Heart, Lung, and Blood Institute Grants R01HL128297 (P.S.), R01HL141080 (P.S.), and R01HL166345; and American Heart Association 18TPA34170588 (P.S.) and 23TPA1074022. T.W.K. was supported by American Heart Association postdoctoral fellowship AHA828786.

## DISCLOSURES

No conflicts of interest, financial or otherwise, are declared by the authors.

## AUTHOR CONTRIBUTIONS

T.P.-S. conceived and designed research; T.W.K., Ay.S., A.M., A.S., R.U., R.K.D., B.S., C.H., O.K., J.T., and T.P.-S. performed experiments; T.W.K., Ay.S., A.M., R.U. and T.P.-S. analyzed data; Ay.S., R.U., G.J.K., and T.P.-S. interpreted results of experiments; R.U., Ay.S., T.W.K., A.S. and T.P.-S. prepared figures; T.P.-S. drafted manuscript; E.M.N. and T.P.-S. edited and revised manuscript; All the authors approved final version of manuscript.
